# Hydrogen Diffusion in Hybrid Perovskites from Exchange NMR

**DOI:** 10.1021/acs.chemmater.4c01498

**Published:** 2024-07-24

**Authors:** Michael A. Hope, Aditya Mishra, Lyndon Emsley

**Affiliations:** † Department of Chemistry, University of Warwick, Coventry CV4 7AL, United Kingdom; ‡ Institut des Sciences et Ingénierie Chimiques, École Polytechnique Fédérale de Lausanne (EPFL), CH-1015 Lausanne, Switzerland

## Abstract

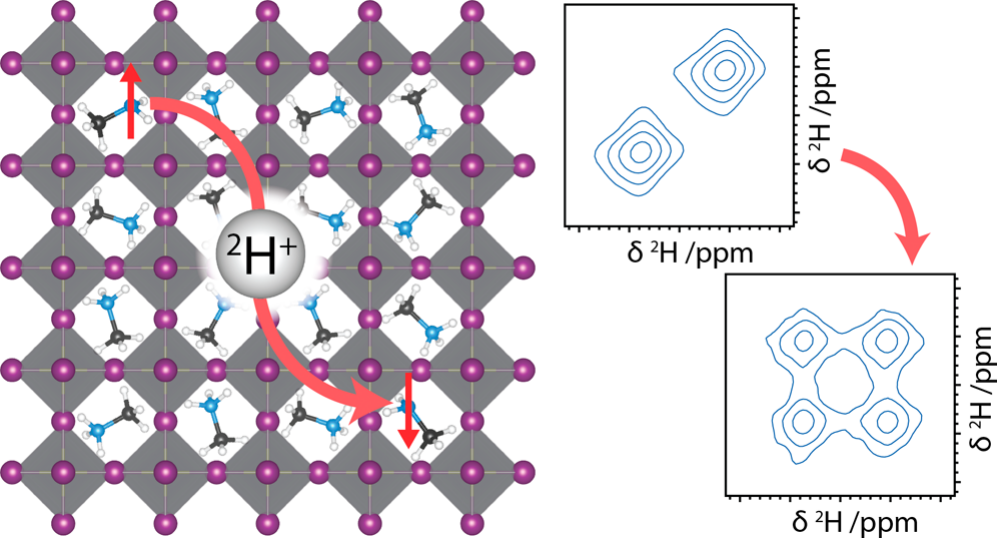

Ion migration is an important phenomenon affecting the performance of hybrid perovskite solar cells. It is particularly challenging, however, to disentangle the contribution of H^+^ diffusion from that of other ions, and the atomic-scale mechanism remains unclear. Here, we use ^2^H exchange NMR to prove that ^2^H^+^ ions exchange between MA^+^ cations on the time scale of seconds for both MAPbI_3_ and FA_0.7_MA_0.3_PbI_3_ perovskites. We do this by exploiting ^15^N-enriched MA^+^ to label the cations by their ^15^N spin state. The exchange rates and activation energy are then calculated by performing experiments as functions of mixing time and temperature. By comparing the measured exchange rates to previously measured bulk H^+^ diffusivities, we demonstrate that, after dissociating, H^+^ ions travel through the lattice before associating to another cation rather than hopping between adjacent cations.

## Introduction

**Figure 1:**
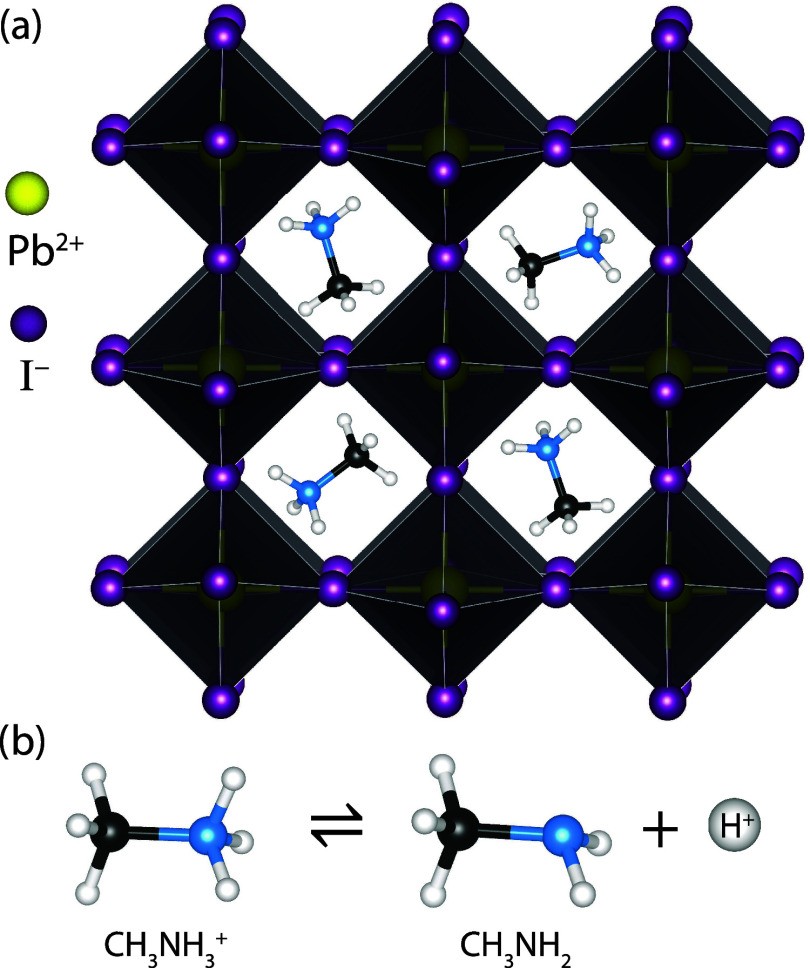
(a) Schematic crystal structure of MAPbI_3_ adapted from ICSD entry 124919.^20^ (b) Deprotonation of MA^+^.

Hybrid lead–halide perovskites are of significant interest as materials for next-generation solar cells, with power conversion efficiencies now exceeding 26% in single-junction devices.^1−4^ These materials have the general formula APbX_3_, where A^+^ is an organic cation such as methylammonium (MA^+^, CH_3_NH_3_^+^) and X^–^ is a halide (Figure 1a). Importantly, the structure is dynamic: in particular, constituent ions can migrate, causing accumulation of ions at the contacts between the perovskite and the electron/hole transport layers in perovskite solar cell devices.^5−7^ This inhibits efficient extraction of charge carriers, causing a loss in photoconversion efficiency that manifests as a current–voltage hysteresis,^6,8−12^ and may adversely affect the long-term efficiency of perovskite solar cells, especially at elevated temperatures.^13,14^ The migration of halide ions is well known for hybrid perovskite materials; however, it has also been shown that the organic cations can deprotonate to generate mobile H^+^ ions and that, in some cases, H^+^ migration can dominate the ionic response (Figure 1b).^15^

H^+^ migration in MAPbI_3_ was first investigated computationally.^16^ Indirect experimental support then came from the temperature-independent ionic conductivity below ∼80 K^17^ and the increased photoluminescence following treatment of MAPbI_3_ films with a polybase.^18^ Long-range H^+^ migration in MAPbI_3_ was later supported directly by isotope-exchange experiments: exposure of MAPbI_3_ single crystals to D_2_O vapor resulted in deuteration of the −NH_3_ moieties of MA^+^ in the bulk, as quantified by solution NMR following dissolution,^15^ and exposure of deuterated MAPbI_3_ thin films to even low vapor pressures of H_2_O resulted in back exchange through the thickness of the film, as determined by vibrational spectroscopy.^19^ Direct atomic-scale evidence of H^+^ mobility is, however, still lacking.

Nuclear magnetic resonance (NMR) spectroscopy is sensitive to dynamic processes across a wide range of time scales,^21,22^ and has been applied extensively to study ionic transport, for example, in solid electrolytes for battery and fuel-cell applications.^22−25^ NMR has also been applied to elucidate various phenomena for hybrid perovskite systems,^26−28^ including ionic transport in MAPbI_3_.^29^ Senocrate et al. used the temperature dependence of the ^127^I nuclear quadrupole resonance line width to deduce mobility of the iodide ions, and on the basis of the temperature-independent ^1^H NMR line width concluded there is no perceptible long-range diffusion of MA^+^;^29^ however, they did not consider the possibility of H^+^ migration.

There are three main classes of NMR experiments that can be used to investigate ionic mobility in the solid state: relaxometry, line width analysis, and exchange spectroscopy (EXSY) experiments.^22^ For the hydrogen nuclei in hybrid perovskites, the *T*_1_ and *T*_2_ relaxation for both ^1^H and ^2^H are dominated by the fast rotational cation dynamics^30−33^ and are insensitive to H^+^ migration. We recently observed evidence for H^+^ migration in MAPbI_3_ to paramagnetic relaxation sinks because the ^2^H *T*_1_ relaxation becomes faster above ∼310 K;^33^ however, this is an indirect probe that is challenging to analyze quantitatively. As for line width analysis, the H^+^ diffusion is too slow to average the ^1^H dipolar broadening (which yields an 8.2 kHz static line width)^29^ and the residual ^2^H quadrupolar coupling in the tetragonal phase cannot be averaged since it is the same for all sites in a given crystallite. Exchange spectroscopy can probe dynamics on longer time scales limited only by the *T*_1_, on the order of 10 s for ^1^H/^2^H in MAPbI_3_. However, exchange spectroscopy requires the nuclei to move between environments with different NMR frequencies, whereas in single-cation perovskites, all of the A-site cations are equivalent.

Here, we use ^15^N-enriched MA^+^ to label the cations by their ^15^N spin state, effectively partitioning the cations into two chemically equivalent but magnetically inequivalent sites. We then measured the exchange of ^2^H^+^ nuclei between these cations in partially deuterated ^15^N-MAPbI_3_ using high-resolution magic angle spinning (MAS) two-dimensional (2D) ^2^H EXSY NMR. ^2^H NMR was chosen over ^1^H NMR for the weaker homonuclear dipolar coupling, which affords narrower resonances and suppressed spin diffusion. We demonstrate unambiguously that ^2^H^+^ ions hop between cations on the time scale of seconds, measure the activation energy for H^+^ exchange, and discuss the implications for the atomic-scale mechanism of H^+^ migration in hybrid perovskites containing MA^+^.

## Results

**Figure 2:**
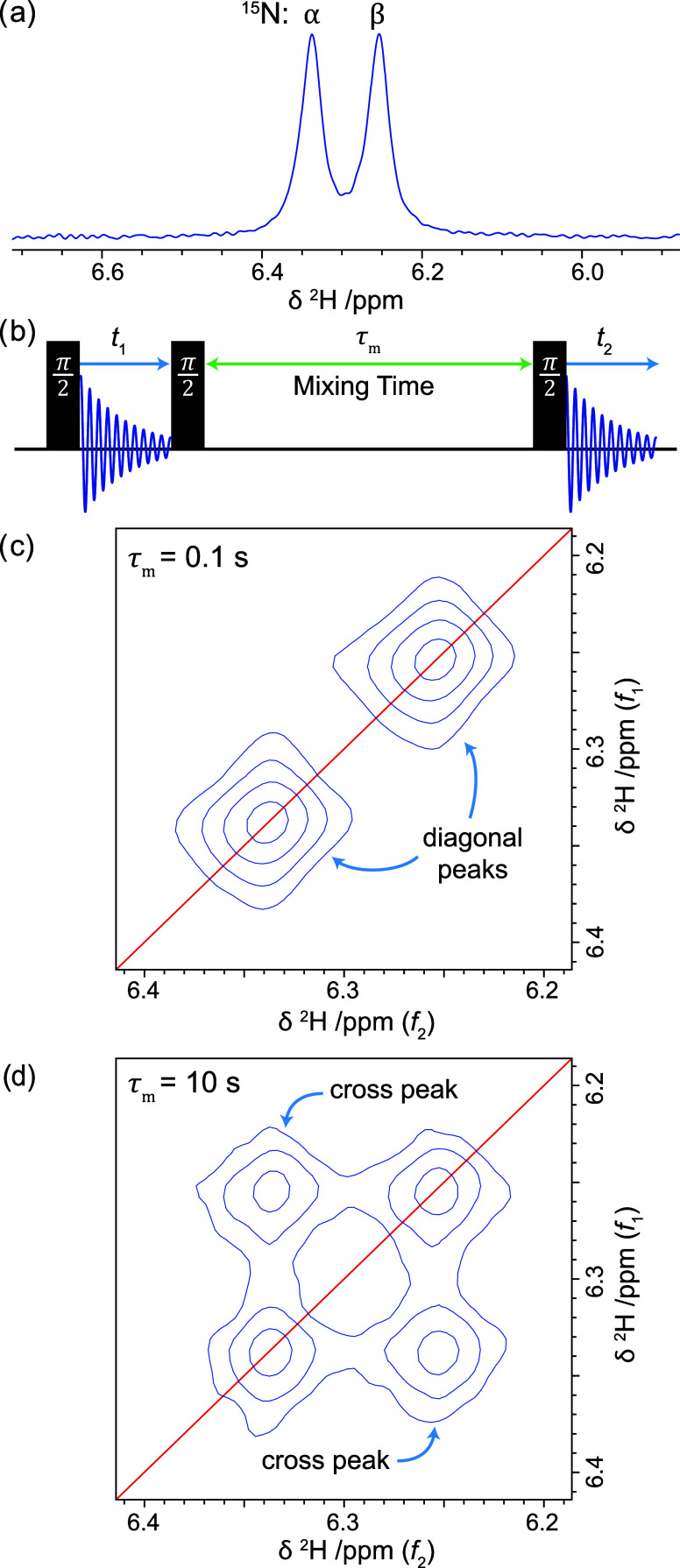
21.1 T (138 MHz) ^2^H NMR spectra of 15% N-deuterated ^15^N-MAPbI_3_ at a sample temperature of 346 K and 20 kHz MAS. (a) 1D spectrum obtained with a single pulse experiment. (b) EXSY pulse sequence. (c, d) Contour plots of 2D EXSY spectra obtained with 0.1 and 10 s mixing times, respectively. The red lines indicate the spectrum diagonal (*f*_1_ = *f*_2_). Experimental details are given below as well as a link to all of the raw data.

Figure 2a shows the 21.1 T ^2^H NMR spectrum of mechanosynthesised 15% N-deuterated ^15^N-MAPbI_3_ (i.e., [CH_3_^15^N(H_0.85_D_0.15_)_3_]PbI_3_) powder at 346 K with MAS at 20 kHz (the deuteration level was measured by ^1^H NMR, Figure S1). MAS yields two sharp peaks, centered at a chemical shift of 6.3 ppm and split by the ^15^N–^2^H *J*-coupling, ^1^*J* = 11.6 Hz. One peak arises from cations with ^15^N in the α (spin-up) state, and the other from cations with ^15^N in the β (spin-down) state. The ^15^N spins relax with a time constant *T*_1_ = 134 s at this temperature (Figure S3); therefore, for shorter times, the ^15^N spin does not spontaneously flip, and the two ^2^H signals correspond to two distinct populations of cations, which are randomly distributed within the sample.

A two-dimensional EXSY experiment (Figure 2b)^34^ correlates frequencies between the first (indirect) evolution period (*t*_1_) and the second (directly detected) evolution period (*t*_2_). Between these two periods, the magnetization is stored for a mixing time (τ_m_). If a spin remains in the same environment during the mixing time, its NMR frequency is the same in *t*_1_ and *t*_2_, leading to a diagonal peak in the 2D frequency-domain spectrum (*f*_1_ = *f*_2_). In contrast, if the spin changes environment during the mixing time, a cross-peak is observed between the frequencies before and after exchange (*f*_1_ ≠ *f*_2_).

Figure 2c shows the ^2^H EXSY spectrum of 15% N-deuterated ^15^N-MAPbI_3_ at 346 K with a mixing time of 0.1 s. This is not long enough for any significant number of ^2^H^+^ ions to hop from one cation to another; therefore, only diagonal peaks are observed. Figure 2d shows the ^2^H EXSY spectrum under the same conditions with a mixing time of 10 s. Now, strong cross-peaks are observed due to exchange of ^2^H spins between ^15^N_α_-MA^+^ and ^15^N_β_-MA^+^ cations (and vice versa) during the mixing time. In fact, the intensities of the cross and diagonal peaks are almost equal, indicating near-complete equilibration of populations during the mixing time. Importantly, the 10 s mixing time is still over an order of magnitude less than the ^15^N *T*_1_ constant.

**Figure 3:**
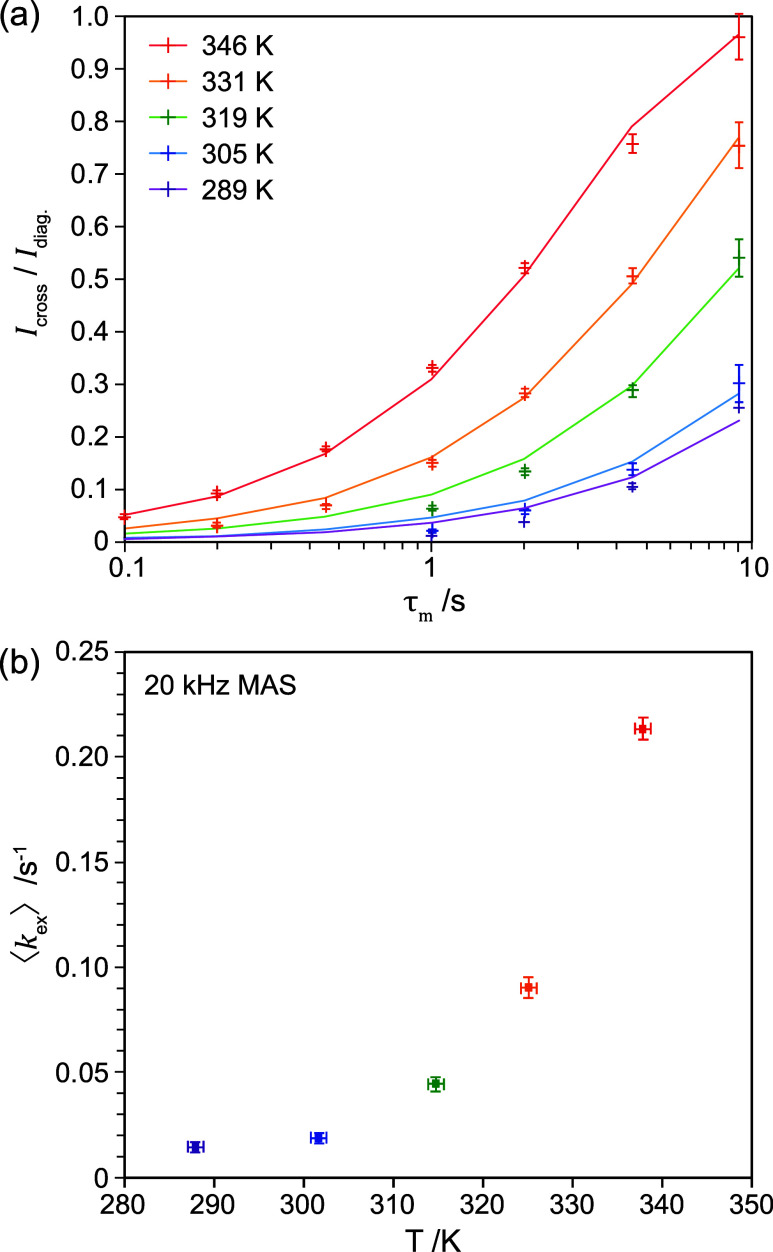
(a) *I*_cross_(τ_m_)/*I*_diag._(τ_m_) in the ^2^H EXSY spectra of 15% N-deuterated ^15^N-MAPbI_3_ as a function of the mixing time for different temperatures and fits to a stretched tanh function (solid lines). The peak intensities were extracted by fitting the 2D spectra. (b) The average exchange rate, ⟨*k*_ex_⟩, as a function of temperature obtained from the fits in panel (a).

To measure the rate of ^2^H exchange between cations, ^2^H EXSY spectra were measured as a function of mixing time, up to τ_m_ = 10 s (this is limited by the ^2^H *T*_1_ ≈ 6 s at 346 K). The cross-peaks build up with a rate *k*_ex_, and all of the peaks decay according to the ^2^H *T*_1_ relaxation; to remove the latter contribution, we consider the ratio of the fitted cross and diagonal peak intensities, which follows a tanh equation dependent only on the exchange rate.^34^ Here, we use a stretched tanh to account for a distribution of exchange rates (most likely due to MAS-induced temperature gradients in the sample):
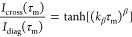
where β < 1 is the stretching exponent. The average value of *k*_ex_ over this distribution is
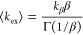
where Γ is the gamma function.^35^Figure 3a shows the ^2^H EXSY buildup at 20 kHz MAS and the corresponding fit to a stretched tanh function. At 346 K, we determine that ⟨*k*_ex_⟩ = 0.214 ± 0.005 s^–1^, corresponding to an exchange time constant of ∼5 s.

To determine the activation energy for ^2^H exchange, EXSY spectra were recorded with variable mixing times as a function of temperature between 298 and 346 K and fitted to a stretched tanh function. For consistency, a stretching exponent of β = 0.8 was used at all temperatures, based on the fitted value at 346 K. Figure 3b shows the measured exchange rate as a function of the temperature. The rate increases with increasing temperature, consistent with an activated process. However, the rate exhibits a nonzero plateau at the lowest temperature studied.

In addition to physical exchange, cross-peaks in EXSY spectra can also arise from spin diffusion due to dipolar coupling between the spins. In these experiments, spin diffusion is largely suppressed by the low gyromagnetic ratio of deuterium, the low ^2^H concentration (15% labeling), and the relatively fast MAS rate (20 kHz), which all reduce the dipolar coupling; nevertheless, spin diffusion can still contribute to the measured exchange. A sample with a higher deuteration level of 90% and a lower MAS rate of 10 kHz exhibits a much higher exchange rate caused by spin diffusion, which masks the physical exchange (Figure S4). Measuring the exchange rate as a function of MAS for both samples at the same temperature shows that the ^2^H spin diffusion depends quadratically on MAS, *k*_SD_ ∝ 1/ν_MAS_^2^ (Figure S5), whereas the rate of physical exchange between cations is unaffected by MAS (note that the measured temperature includes any frictional heating). Therefore, to determine the contribution of spin diffusion to the measured exchange rate, ^2^H EXSY spectra were measured at both 10 and 20 kHz MAS at each temperature for the 15% deuterated sample (Figure S6).

**Figure 4:**
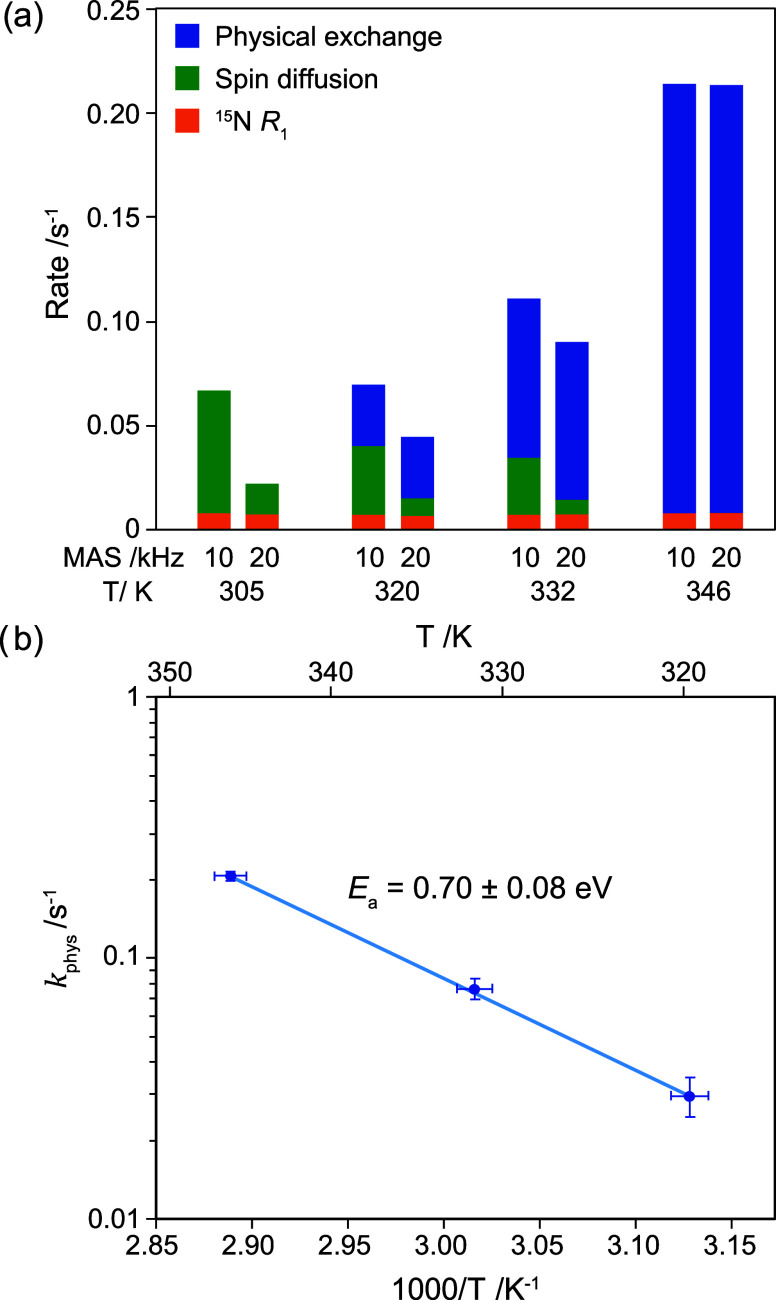
(a) Breakdown of the three contributions to the observed ^2^H exchange rate as a function of temperature at 10 and 20 kHz MAS rate. See Table S1 for uncertainties which are not included in the figure. (b) Arrhenius plot of the physical exchange rate of ^2^H between MA^+^ cations as a function of temperature.

The total measured exchange rate between ^15^N_α_-MA^+^ and ^15^N_β_-MA^+^ cations (*k*_ex_) is thus composed of three components: physical exchange of ^2^H^+^ ions between cations, ^2^H–^2^H spin diffusion, and ^15^N spin flips:
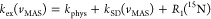
where *R*_1_(^15^N) = 1/*T*_1_(^15^N). Using the dependence *k*_SD_ ∝ 1/ν_MAS_^2^, the data at 10 and 20 kHz MAS can be combined to eliminate the spin diffusion contribution:
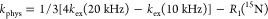


Figure 4a shows a breakdown of these three contributions as a function of temperature at 10 and 20 kHz MAS rates (data in Table S1). The spin diffusion rate decreases with increasing temperature, which is ascribed to the narrowing of the zero-quantum line width;^36,37^ this can be more clearly seen for the highly deuterated sample (Figure S4). At 305 K (and below), spin diffusion dominates, and the contribution of physical exchange cannot be accurately distinguished.

Eliminating the contributions of spin diffusion and ^15^N relaxation unambiguously demonstrates physical exchange of H^+^ ions between MA^+^ cations in MAPbI_3_, on the time scale of seconds. This physical exchange follows an Arrhenius dependence with an activation energy of *E*_a_ = 0.70 ± 0.08 eV (Figure 4b and Table S3). Ceratti et al.^15^ previously determined an activation energy of *E*_a_ = 0.49 eV for macroscopic diffusion of ^2^H into single crystals of protonated MAPbI_3_ exposed to D_2_O vapor. In contrast, we have measured the activation energy for the atomic-scale process of ^2^H migration from one cation to another (see below for further discussion).

**Figure 5:**
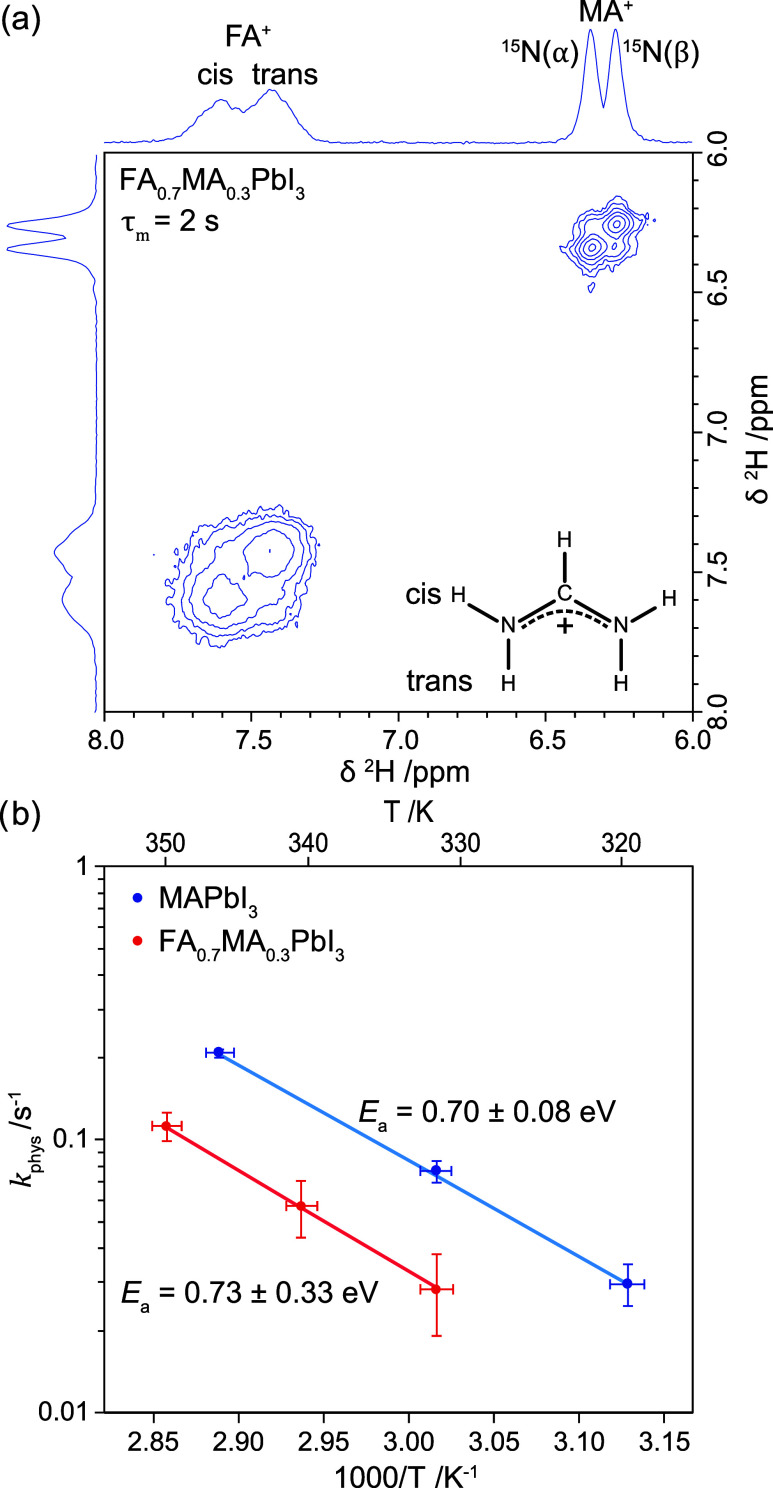
(a) ^2^H EXSY spectrum of FA_0.7_MA_0.3_PbI_3_, with ^15^N-labeled MA^+^, ∼20% deuteration of the MA^+^ –NH_3_ hydrogens, and ∼7% deuteration of the FA^+^–NH_2_ hydrogens, at 21.1 T, 20 kHz MAS, and 340 K, with 2 s of mixing time. The recycle delay was 7.5 s. (b) Arrhenius plot of the MA^+^–MA^+^ physical exchange rate in MAPbI_3_ and FA_0.7_MA_0.3_PbI_3_ as measured by ^2^H EXSY spectra with variable mixing times.

Next, we extended the method to mixed MA^+^–FA^+^ perovskites (FA^+^ = formamidinium, CH(NH_2_)_2_^+^), as typically used in modern perovskite solar cells.^2,3^Figure 5a shows the ^2^H EXSY spectrum of mechanosynthesised FA_0.7_MA_0.3_PbI_3_ at 340 K with ^15^N-labeled MA^+^, ∼20% deuteration of the MA^+^–NH_3_ hydrogens, and ∼7% deuteration of the FA^+^–NH_2_ hydrogens. As for pure MAPbI_3_, the MA^+^ signal is split by the ^15^N *J*-coupling, whereas, for FA^+^, the inequivalent cis and trans −NH_2_ positions can be resolved.^33^ With a mixing time of 2 s, exchange between MA^+^ cations with different ^15^N spin states is again observed; however, there is no discernible exchange between MA^+^ and FA^+^. This is consistent with the lower acidity of the FA^+^ cation,^38,39^ meaning it is harder to deprotonate. Furthermore, if there was appreciable hydrogen exchange between MA^+^ and FA^+^, the deuteration levels would be expected to equalize, which was not observed. Cross-peaks between the *cis* and *trans* FA^+^ signals can also be distinguished (*k*_ex_ ≈ 0.1 s^–1^), which is ascribed to the slow restricted rotation of the −NH_2_ group, since physical exchange would also give cross-peaks with MA^+^ and spin diffusion should be negligible for the 7% −NH_2_ deuteration level.

To compare the hydrogen migration in FA_0.7_MA_0.3_PbI_3_ and MAPbI_3_, ^2^H EXSY spectra were measured as a function of mixing time and temperature, focusing on the MA^+^ signals (Figure S7 and Table S2). The physical exchange rate was then calculated by subtracting the ^15^N relaxation; based on the low MA^+^ content and lack of FA^+^–MA^+^ cross-peaks, spin diffusion is assumed to be negligible. Figure 5b compares the MA^+^–MA^+^ physical exchange in FA_0.7_MA_0.3_PbI_3_ and MAPbI_3_. The low signal-to-noise ratio due to the low MA^+^ content results in appreciable uncertainty, but the Arrhenius behavior expected for activated physical exchange is still clear. The exchange rates are lower for FA_0.7_MA_0.3_PbI_3_ by a factor of ∼3, which is ascribed to the 3.3× lower concentration of MA^+^. The fitted activation energy (*E*_a_ = 0.73 ± 0.33 eV) is the same as that for MAPbI_3_ (*E*_a_ = 0.70 ± 0.08 eV) within error (Table S3). This suggests the same rate-limiting step in each case, most likely the deprotonation of MA^+^.

We now compare the rate of atomic-scale exchange of ^2^H^+^ ions between MA^+^ cations, as measured here, and the previously measured diffusion rates for bulk exchange of ^1^H/^2^H in MAPbI_3_ single crystals and thin films (Table 1).^15,19^ The range of reported values covers several orders of magnitude, as has been discussed previously,^40,41^ which could reflect differences between the H^+^ diffusivity in thin films and large single crystals, as well as differences in hydration, solvation, and surface passivation. These factors are also likely to be different for the microcrystalline powders studied here; nevertheless, significant insight can be gained from a semiquantitative comparison.

**Table 1:** Previous Literature Room Temperature H^+^ Diffusivities (*D*) for Bulk ^1^H/^2^H Exchange,^15,19^ the Average Jump Distance, *x*, Required to Give These Diffusivities if Mediated by 3D Jumps at a Rate *k* = 0.0094 s^–1^ (i.e., as Measured Here), and the Estimated H^+^ Vacancy Concentration, [CH_3_NH_2_], That Would be Required for the Average Jump to Be by This Distance

	Sadhu et al., thin films	Ceratti et al., single crystals
*D* (298 K)/cm^2^ s^–1^	1.5 × 10^–15^^19^	5.2 × 10^–10^^15^
*x*/nm	9.8	5800
[CH_3_NH_2_]/cm^–3^	1.8 × 10^19^	6.6 × 10^12^

Extrapolating the Arrhenius behavior in Figure 4b to 298 K gives a physical exchange rate of 0.0047 s^–1^ for MAPbI_3_. This is the rate of ^2^H^+^ exchange between MA^+^ cations with opposite ^15^N spin states; the total rate of ^2^H^+^ exchange is therefore double this, *k* = 0.0094 s^–1^. If H^+^ diffusion is mediated by H^+^ exchange between MA^+^ cations at a rate *k*, the diffusivity in three dimensions (3D) is given by
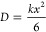
where *x* is the distance between the exchanging MA^+^ cations.^42^ Assuming exchange between adjacent cations (*x* = 6.3 Å) gives a diffusivity of *D* = 6.2 × 10^–18^ cm^2^ s^–1^, which is much lower than observed experimentally. This rules out a direct vacancy mechanism, where hops occur between adjacent cation sites for H^+^ diffusion in MAPbI_3_.

Instead, H^+^ diffusion could occur via an interstitial mechanism. In this case, an H^+^ ion dissociates from an MA^+^ cation and then travels through the lattice before associating with another MA, giving a larger effective jump distance, *x*. Possible migration pathways of interstitial H^+^ have previously been identified computationally.^16,43^Table 1 shows the average jump distance required to give the measured diffusivities if mediated by 3D jumps at the exchange rate determined here: *x* = 10 nm and *x* = 6 μm for the data of Sadhu et al.^19^ and Ceratti et al.,^15^ respectively. H^+^ ions traveling a significant distance through the lattice is consistent with neutral deprotonated CH_3_NH_2_ molecules being a minority species in the bulk (i.e., a low H^+^ vacancy concentration),^44^ since the migrating H^+^ must find a deprotonated MA to reprotonate (i.e., a vacancy to fill). By simulating H^+^ ions undergoing a random walk as a function of H^+^ vacancy concentration (see the Experimental Section), the concentration required for the calculated jump distances can be estimated (Table 1). A defect concentration in the range 10^12^ – 10^19^ cm^–3^ is reasonable in hybrid perovskite systems.^45^ Although a hop distance of 6 μm and a vacancy concentration of 7 × 10^12^ cm^–3^ may be possible in the single crystals (>1 mm) studied by Ceratti et al.,^15^ it seems more likely that the diffusivity in their samples is higher than in the samples studied here, leading to an overestimate of *x*.

Another possible mechanism is for H^+^ ions to associate or exchange with an H_2_O molecule, which then diffuses through the lattice before exchanging with another cation. Partial hydration of MAPbI_3_ is known to be possible,^46−48^ and diffusion of H_2_O has previously been reported.^49^ However, we note that we did not observe any evidence of H_2_O by ^1^H or ^2^H NMR, implying a low concentration in the samples studied here (undergoing magic angle spinning using nitrogen gas flows; in contrast, the experiments of Ceratti et al.^15^ were performed at 45% relative humidity). The lower activation energy measured by Ceratti et al.^15^ (0.49 vs 0.70 eV here) may correspond to the water diffusion rather than the MA^+^ deprotonation and exchange measured by our NMR experiments.

Whether the H^+^ ions travel as H^+^ interstitials or via molecules, our analysis demonstrates that H^+^ ions must move through the lattice between dissociating from one cation and associating with another in order to reconcile the atomic-scale exchange rate and the bulk diffusivity. This mechanistic insight adds to the growing picture of complex ion dynamics in these materials, facilitating the development of strategies to mitigate ionic motion and enhance the performance of photovoltaic devices.

## Conclusions

In conclusion, we have demonstrated the atomic-scale physical exchange of ^2^H^+^ ions between MA^+^ cations in MAPbI_3_ and FA_0.7_MA_0.3_PbI_3_ on the time scale of seconds using ^2^H exchange NMR. This was achieved by labeling the MA^+^ cations according to the ^15^N spin state, effectively splitting the cations into two inequivalent populations. The activation energy for exchange was extracted by performing experiments as a function of the mixing time and temperature, yielding *E*_a_ = 0.70 ± 0.08 eV in MAPbI_3_ and *E*_a_ = 0.73 ± 0.33 eV in FA_0.7_MA_0.3_PbI_3_. In contrast, no physical exchange was observed with FA^+^, in line with its lower acidity. Finally, we compared the ^2^H exchange rate with the bulk H^+^ diffusivity, demonstrating that after dissociating, H^+^ ions travel through the lattice before associating with another cation rather than hopping between adjacent cations.

## Experimental Section

### Sample Preparation

MAPbI_3_ and FA_0.7_MA_0.3_PbI_3_ were prepared from CH_3_^15^ND_3_I (99% ^15^N, >97% D, CortecNet), natural abundance FAI (Sigma-Aldrich), and PbI_2_ (Sigma-Aldrich) by mechanosynthesis with a Retsch MM-200 shaker mill at 25 Hz for 30 min, using 150 mg of precursors in a 2 mL polypropylene Eppendorf and one stainless-steel ball (Ø 4 mm). Samples were annealed for 15 min at 60 °C (MAPbI_3_) or 120 °C (FA_0.7_MA_0.3_PbI_3_). The deuteration level of MAPbI_3_ was reduced by leaving in air overnight to give a balance of sufficient sensitivity but suppressed ^2^H–^2^H spin diffusion. Phase purity was checked for a natural abundance sample of MAPbI_3_ by X-ray diffraction using a Bruker D8 Discover Vario diffractometer with Cu Kα radiation (Figure S9). Samples were packed into 3.2 mm outer-diameter rotors, half-filling them, before adding a small spacer of PTFE tape and a layer of Pb(NO_3_)_2_ as an in situ NMR thermometer.

### NMR Spectroscopy

Solid-state NMR experiments were performed on a 21.1 T Bruker AVANCE NEO spectrometer using a low-temperature MAS probe for 3.2 mm rotors. Spectra were acquired at a 20 kHz MAS unless otherwise stated. Sample heating was achieved by heating the bearing, drive, and variable temperature gas flows. Sample cooling was achieved by flowing the variable temperature gas through a liquid-nitrogen heat exchanger. The sample temperature was measured using the temperature-dependent ^207^Pb shift of Pb(NO_3_)_2_.^50^ Deuteration levels were measured by integrating the ^1^H NMR spectrum acquired with a Hahn echo and a 30 s recycle delay. The ^15^N *T*_1_ was measured with a saturation recovery sequence and 10 kHz ^1^H WALTZ decoupling during acquisition. 138 MHz ^2^H EXSY spectra were recorded without ^1^H decoupling, with 4 scans, a recycle delay of 5 s, and between 20 and 30 steps of 20 ms in the indirect dimension (the spectrum including both FA^+^ and MA^+^ used 120 steps of 3.33 ms). 1 Hz of exponential line-broadening was applied in each dimension prior to Fourier transform. The magic angle and z-shim were adjusted to maximize the ^2^H peak intensity for each sample.

### Data Analysis

2D spectra were deconvoluted using dmfit,^51^ and the cross/diagonal ratio was calculated as the average cross-peak intensity divided by the average diagonal peak intensity: (*I*_αβ_ + *I*_βα_)/(*I*_αα_ + *I*_ββ_). The uncertainty in the ratio was calculated from the spectral noise level and peak intensities. The exchange buildup rates were fitted to a (stretched) tanh function using Matlab. For MAPbI_3_, the stretching parameter was fixed to β = 0.8 based on the optimum value at 346 K. For FA_0.7_MA_0.3_PbI_3_, the best fit was with β = 1. The uncertainties in the fitted buildup rates were calculated by Monte Carlo analysis: the fitted cross/diagonal ratios were changed randomly according to their uncertainty to give a distribution of fitted buildup rates, the standard deviation of which is the uncertainty. The activation energy for exchange and its uncertainty were similarly calculated by fitting the exchange rates as a function of temperature to an Arrhenius equation and using Monte Carlo analysis based on the calculated uncertainty in the exchange rates and a 1 K uncertainty in the temperature. H^+^ diffusion was simulated in Matlab by considering a 3D random walk on a cubic lattice in the presence of an equally spaced cubic array of vacancy sites at a given concentration; for each concentration, 2000 trials were performed, and the jump distance was taken as the mean distance to the first vacancy encountered.

## Data Availability

Raw and processed NMR data are available at DOI: 10.5281/zenodo.10684981 with a CC-BY-4.0 (Creative Commons Attribution-ShareAlike 4.0 International) license.

## Supporting Information Available

^1^H, ^2^H, and ^15^N NMR spectra. Tables of ^2^H exchange rates as a function of temperature and Arrhenius analysis (PDF)
