# Supplementary material for: Hydrogen Diffusion in Hybrid Perovskites from Exchange NMR
Source: Chem Mater. 2024 Jul 24;36(15):7525–32. doi: 10.1021/acs.chemmater.4c01498 (PMC11325541; doi:10.1021/acs.chemmater.4c01498)
Supplement: Supplementary file 1 — cm4c01498_si_001.pdf [file cm4c01498_si_001.pdf]

Supporting Information

Hydrogen Diffusion in Hybrid Perovskites from Exchange NMR

Michael A. Hope,[1,*] Aditya Mishra,[2,†] Lyndon Emsley[2,*]

1. Department of Chemistry, University of Warwick, Coventry, CV4 7AL, United Kingdom
2. Institut des Sciences et Ingénierie Chimiques, École Polytechnique Fédérale de Lausanne (EPFL), CH-1015 Lausanne, Switzerland

† present address: Francis Bitter Magnet Laboratory, Massachusetts Institute of Technology, Cambridge, MA 02139, USA

* michael.hope.1@warwick.ac.uk, lyndon.emsley@epfl.ch

Raw and processed NMR data are available at DOI: 10.5281/zenodo.10684981 with a CC-BY-4.0 (Creative Commons Attribution-ShareAlike 4.0 International) license.

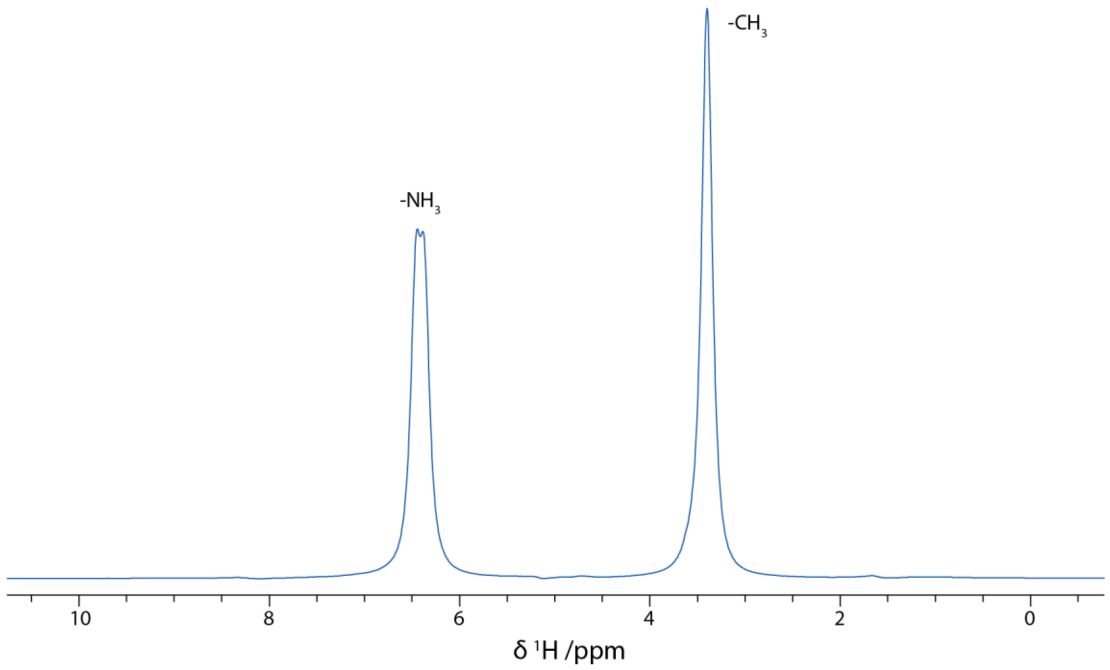

Figure S1: ^{1}H NMR spectrum of 15% N-deuterated ^{15}N-labelled $MAPbI_3$ at 21 T, 20 kHz MAS, and 305 K. The integrated intensity of the $-NH_3$ signal is 85% that of $-CH_3$, indicating 15% deuteration. $^{1}J(^{1}H–^{15}N)$ = 83 Hz.

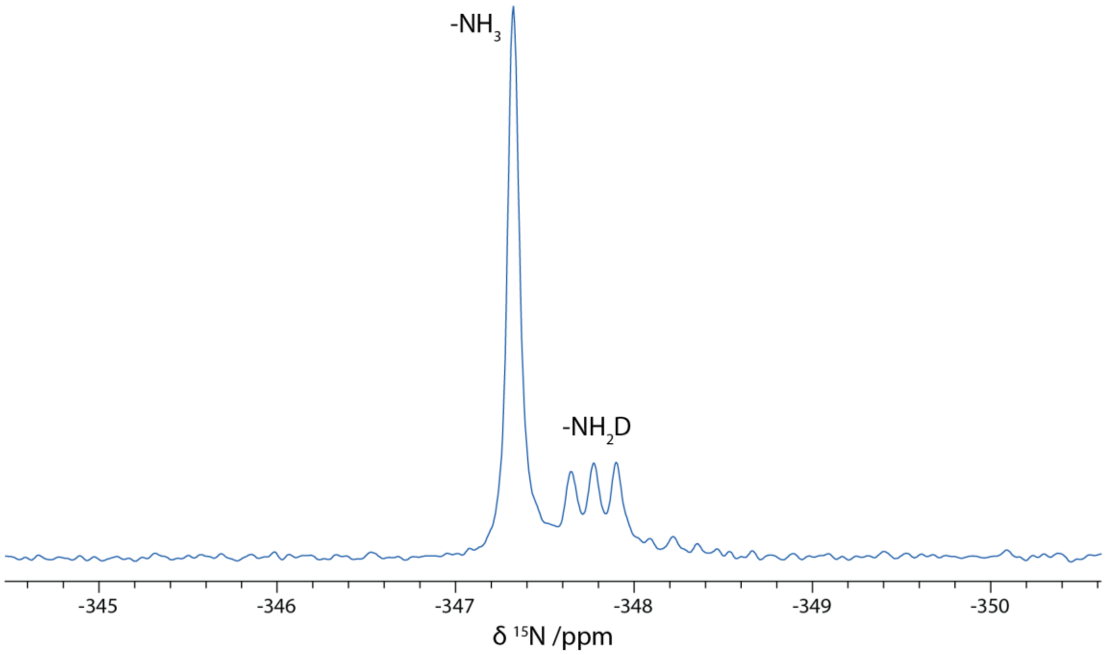

Figure S2: $^{15}N\{^{1}H\}$ spectrum of 15% N-deuterated ^{15}N-$MAPbI_3$ at 21.1 T, 20 kHz MAS, and 346 K. Due to the isotope effect, the $-NH_3$ and $-NH_2D$ signals can be resolved. Their relative intensities are approximately consistent with the expected binomial distribution for 15% deuteration. The $^{1}J(^{2}H–^{15}N)$ = 11.6 Hz coupling gives a 1:1:1 triplet.

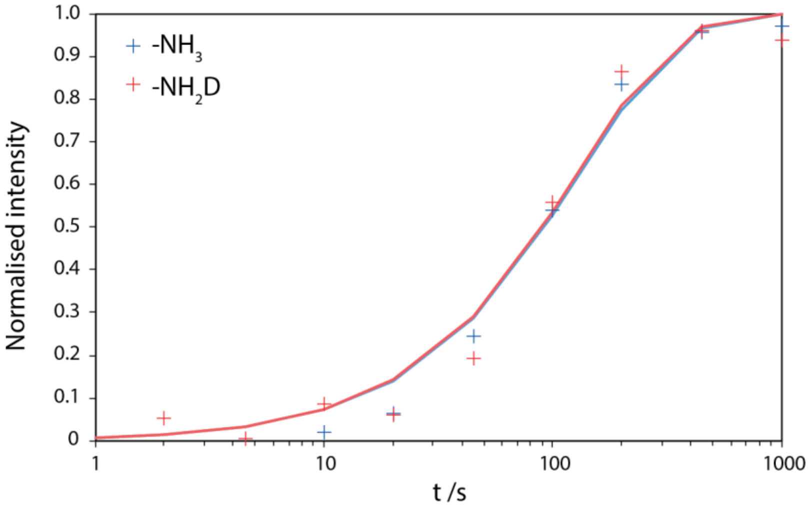

Figure S3: ^{15}N saturation recovery experiment for 15% N-deuterated ^{15}N-$MAPbI_3$ at 21 T, 20 kHz MAS, and 346 K, with low-power ^{1}H decoupling during acquisition. The resolved $-NH_3$ and $-NH_2D$ signals (Figure S2) have indistinguishable relaxation constants of $T_1(-NH_3)$ = 134 ± 10 s and $T_1(-NH_2D)$ = 130 ± 16 s.

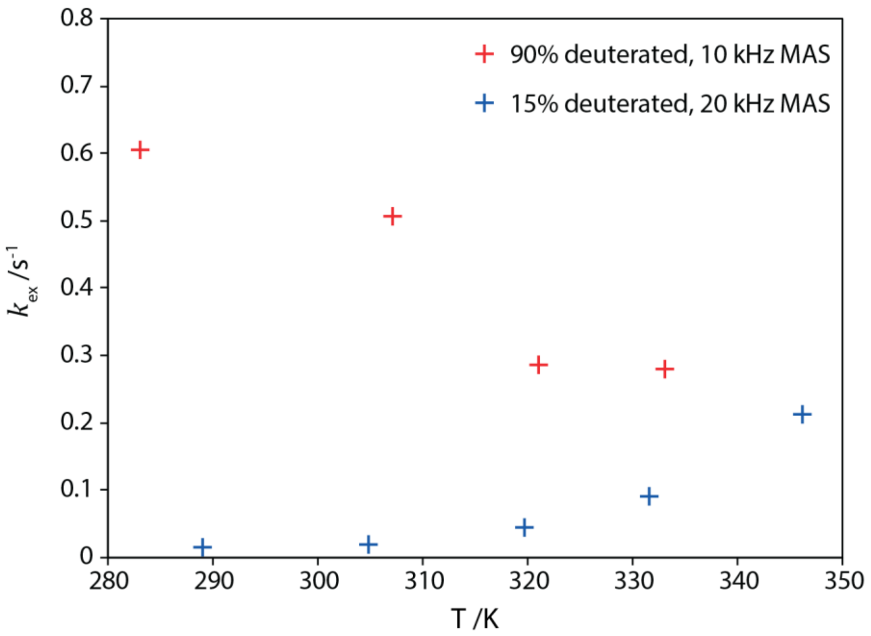

Figure S4: Fitted ^{2}H exchange rates from variable-mixing-time EXSY spectra of N-deuterated ^{15}N-$MAPbI_3$ as a function of temperature, for different deuteration levels. For low deuteration and higher MAS rate, the exchange is dominated by physical exchange, which increases with increasing temperature. For high deuteration and lower MAS rate, the exchange is dominated by spin diffusion, which decreases with increasing temperature, indicating narrowing of the zero-quantum linewidth.

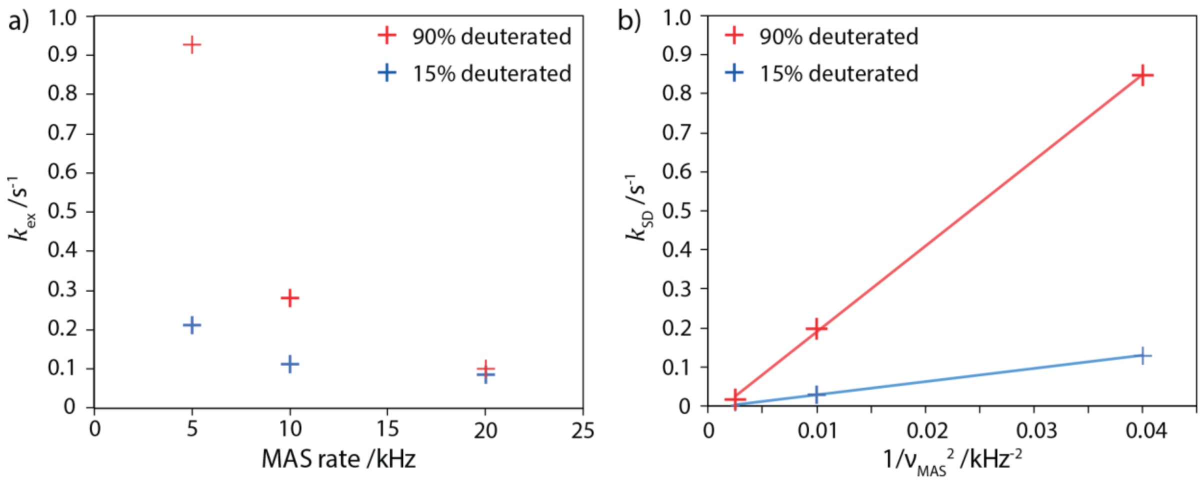

Figure S5: a) Fitted ^{2}H exchange rates from variable-mixing-time EXSY spectra of N-deuterated ^{15}N-$MAPbI_3$ as a function of MAS rate at 319 K for different levels of deuteration. The contribution of spin diffusion to the exchange rate was determined by assuming that the physical diffusion rate is the same for both samples at all MAS rates (fitted as 0.082 s^{-1}), and that the spin diffusion rates of the two samples are related by the same multiplicative factor at each MAS rate (fitted as 6.49, similar to the ratio of the deuteration levels). b) The fitted spin diffusion rates as a function of the inverse square of the MAS rate, showing a linear dependence for both samples. This is ascribed to narrowing of the ^{2}H resonances (Figure S6), and hence of the zero-quantum linewidth.[36,37]

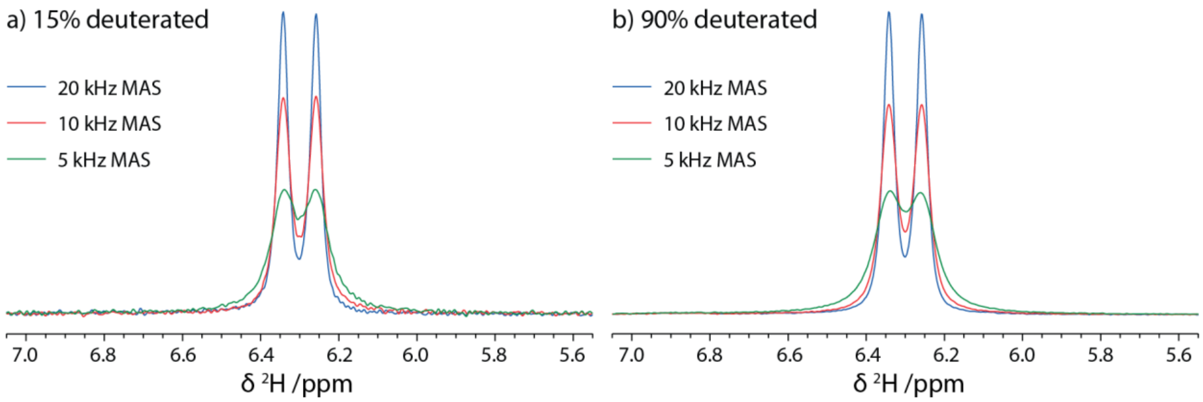

Figure S6: ^{2}H NMR spectra of a) 15% and b) 90% N-deuterated ^{15}N-MAPbI$_3$ as a function of MAS rate at 319 K. With increasing MAS rate, the resonances narrow, resulting in reduced overlap and suppressed spin diffusion.

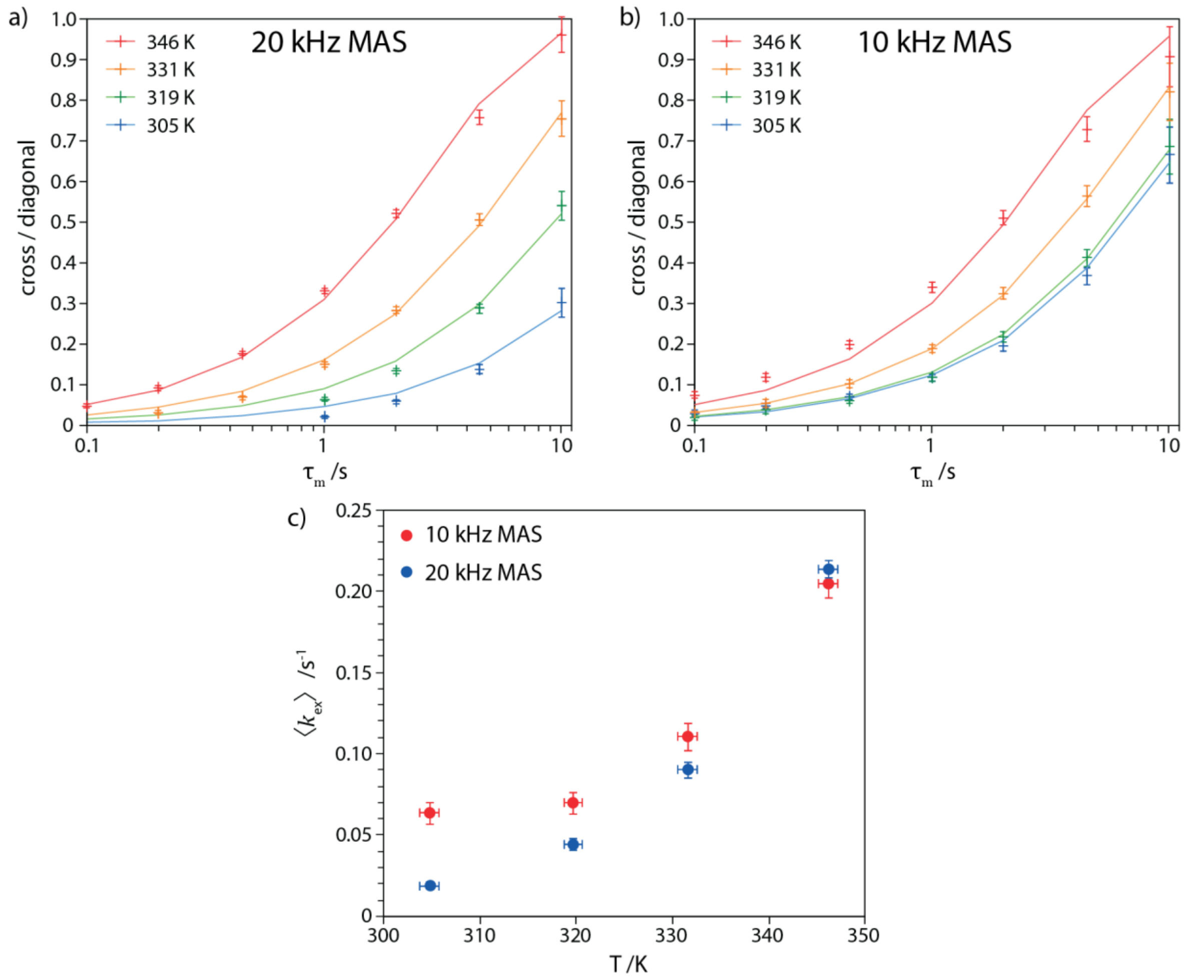

Figure S7: a,b) Build-up of the cross/diagonal peak intensity ratio in the ^{2}H EXSY spectra of 15% N-deuterated ^{15}N-MAPbI$_3$ as a function of mixing time for different temperatures and (a) 20 kHz MAS, (b) 10 kHz MAS, as well as fits to a stretched tanh function (solid lines). c) The average exchange rate, $\langle k_{\mathrm{ex}} \rangle$, as a function of temperature for the fits in (a,b).

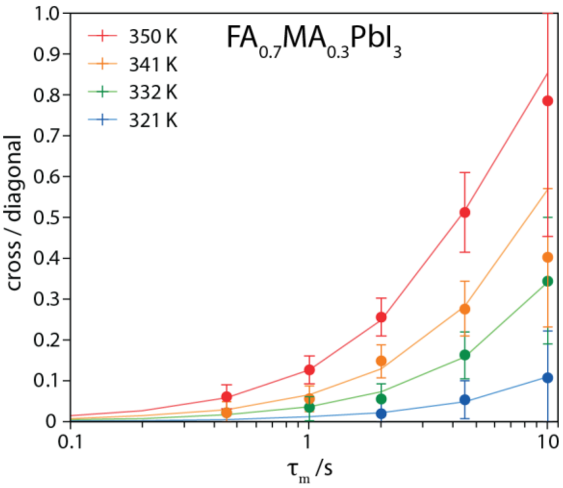

Figure S8: Build-up of the cross/diagonal peak intensity ratio of the ^{15}N-coupled MA^+ peaks in the ^{2}H EXSY spectra of $FA_{0.7}(^{15}N\text{-}MA)_{0.3}PbI_3$ at 20 kHz MAS as a function of mixing time for different temperatures, as well as fits to a tanh function (solid lines).

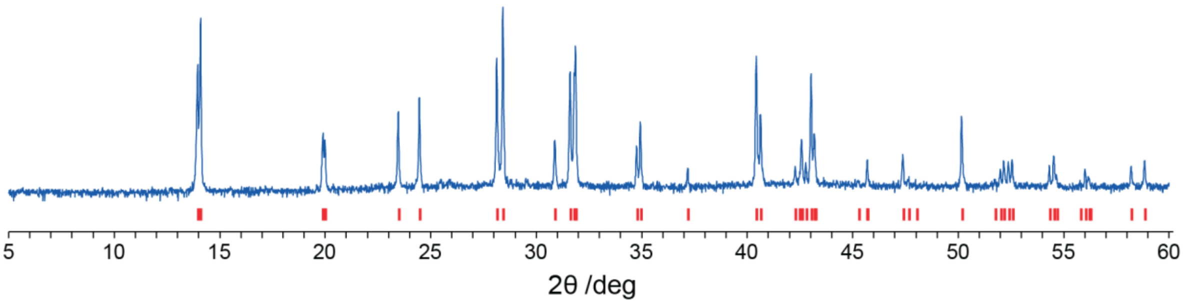

Figure S9: X-ray diffraction pattern of a mechanosynthesised sample of natural abundance $MAPbI_3$ recorded with Cu Kα radiation. The red lines are the calculated reflections for $MAPbI_3$ based on ICSD entry 124919.

Table S1: ^{2}H–^{2}H exchange data for 15% N-deuterated ^{15}N-$MAPbI_3$.

T /K	$\langle k_{ex}\rangle$/s^{-1} [a]		$k_{phys} + R_1(^{15}N)$ /s^{-1} [b]	$R_1(^{15}N)$ /s^{-1}	k_{phys} /s^{-1}
	20 kHz MAS	10 kHz MAS			
305	0.019 ± 0.002	0.064 ± 0.007	0.004 ± 0.004	0.0071 ± 0.0007	~0
320	0.044 ± 0.003	0.070 ± 0.007	0.036 ± 0.005	0.0065 ± 0.0010	0.030 ± 0.005
332	0.090 ± 0.005	0.110 ± 0.009	0.083 ± 0.007	0.0070 ± 0.0008	0.076 ± 0.007
346	0.214 ± 0.005	0.205 ± 0.009	0.214 ± 0.008	0.0077 ± 0.0009	0.206 ± 0.008

a) Fitted ^{2}H–^{2}H exchange rate, calculated from the stretched tanh function by $\langle k_{ex}\rangle = \frac{k_\beta \beta}{\Gamma(1/\beta)}$.

b) Calculated by eliminating spin-diffusion from the measured exchange rate at the two spinning speeds: $\frac{1}{3}[4k_{ex}(20\text{ kHz}) - k_{ex}(10\text{ kHz})]$.

Table S2: Fitted ^{2}H–^{2}H exchange rate for MA^+ in $FA_{0.7}MA_{0.3}PbI_3$ and the calculated physical exchange rate between MA^+ cations.

T /K	k_{ex} /s^{-1}	k_{phys} /s^{-1} [a]
321	0.011 ± 0.009	~0
332	0.036 ± 0.014	0.029 ± 0.014
341	0.064 ± 0.013	0.057 ± 0.013
350	0.119 ± 0.038	0.112 ± 0.038

a) Calculated by assuming the spin-diffusion contribution is negligible, and a temperature-independent ^{15}N R_1 = 0.007 s^{-1}

Table S3: Fitted Arrhenius behaviours for the physical exchange of $^2H^+$ between $^{15}N_\alpha$-MA^+ and $^{15}N_\beta$-MA^+, $k_{phys} = k_0 \exp(-E_a/k_B T)$, where k_B is Boltzmann's constant. Note that the exchange rate of $^2H^+$ between any cations, regardless of spin state, has double the value of k_0.

	$MAPbI_3$	$FA_{0.7}MA_{0.3}PbI_3$
$\log(k_0/s^{-1})$	9.6 ± 1.2	9.6 ± 5.0
E_a /eV	0.70 ± 0.08	0.73 ± 0.33
